# Fiber Optic Temperature Sensor System Using Air-Filled Fabry–Pérot Cavity with Variable Pressure

**DOI:** 10.3390/s23063302

**Published:** 2023-03-21

**Authors:** Hasanur R. Chowdhury, Ming Han

**Affiliations:** Electrical and Computer Engineering Department, Michigan State University, East Lansing, MI 48824, USA

**Keywords:** Fabry–Pérot interferometer, fiber optic sensor, temperature measurement, resolution

## Abstract

We report a high-resolution fiber optic temperature sensor system based on an air-filled Fabry–Pérot (FP) cavity, whose spectral fringes shift due to a precise pressure variation in the cavity. The absolute temperature can be deduced from the spectral shift and the pressure variation. For fabrication, a fused-silica tube is spliced with a single-mode fiber at one end and a side-hole fiber at the other to form the FP cavity. The pressure in the cavity can be changed by passing air through the side-hole fiber, causing the spectral shift. We analyzed the effect of sensor wavelength resolution and pressure fluctuation on the temperature measurement resolution. A computer-controlled pressure system and sensor interrogation system were developed with miniaturized instruments for the system operation. Experimental results show that the sensor had a high wavelength resolution (<0.2 pm) with minimal pressure fluctuation (~0.015 kPa), resulting in high-resolution (±0.32 ℃) temperature measurement. It shows good stability from the thermal cycle testing with the maximum testing temperature reaching 800 ℃.

## 1. Introduction

Fiber optic sensors have gained remarkable popularity due to their many advantages, such as light weight, small size, immunity to electromagnetic interference, compatibility with harsh environments, and multiplexed sensing capability. Therefore, these sensors have been studied extensively for the measurement of a wide range of physical and chemical parameters, including temperature, pressure, strain, displacement, salinity, corrosion in various applications [1,2,3,4].

Temperature is a key parameter that needs to be monitored and controlled precisely in a wide range of applications, including industrial, chemical, structural, biomedical, and environmental aspects. For the last three decades, various techniques have been developed regarding fiber optic temperature sensors such as fiber-Bragg grating (FBG)-based sensors [5], tapered fiber [6], waveguide coupling devices using surface plasmon resonance [7], fiber ring laser demodulation [8], modified fibers (panda fibers [9], multicore fiber [10]), and interferometer-based sensors [11,12,13,14,15,16,17,18,19,20,21,22,23,24,25,26]. Among these, temperature sensors based on Fabry–Pérot (FP) interferometers have proved to be an attractive option because of their additional advantages of simple structure and easy fabrication. They utilize the Fresnel reflections from two parallel reflectors that form the cavity that interfere due to different optical path lengths, nL, where n and L are the refractive index of the material and the cavity length, respectively, both of which are dependent on temperature. Nowadays, different types of FP interferometers are deployed, in terms of their composition and fabrication process. For example, interferometers can belong to the following types (a) fiber-tip: SMF-microfiber [11], SMF-polyvinyl alcohol [12]; (b) with diaphragm: FBG-graphene [13,14], MMF-silicon [15], FBG-fused silica [16]; (c) without diaphragm [17]; (d) polymer materials [18]; (e) inline microcavities [19,20]; (e) multiplexed sensors [21]; (f) microcantilever [22], polished materials [27]. However, each type of sensor has some drawbacks, which limit their application. For example, sensors based on FBGs are prone to strain cross-sensitivity [5]. To overcome this drawback, some studies implement techniques for simultaneous measurement of temperature and pressure or other perturbations [13,20,23]. However, these sensors mostly require calibration due to their inherent FP cavity length change during interrogation. Additionally, for multiple-parameter measurement, many of these works utilize an FBG along with an FP Interferometer sensor, where the lower sensitivity of the FBG to temperature (~10 pm/°C) affects the accuracy of measurements at high temperature [13,23]. Many researchers have used cavities formed by a tip composed of graphene or other materials as diaphragm or cantilever. These materials have large values of thermal expansion coefficient (TEC) and thermo-optic coefficient (TOC) compared to their glass material counterparts, resulting in higher temperature resolution. However, the fabrication process often requires complex MEMS techniques [15,22], chemical processing [12,13,14], or high-power laser ablation [16], which is expensive, time consuming, and lacking in reproducibility. Additionally, these sensors show nonlinearity at high temperature because the composite structure’s TOC becomes a complex function of temperature, resulting in a limited operation range [18]. Researchers have also studied various methods for demodulation of sensor’s spectra. These FP interferometer sensors [24,25,26] are mainly based on dual cavity, where the composite wavelength signal is demodulated by using FFT with various algorithms such as white light interferometry [28], wavelet phase extraction [29], wavelet transform and polarized low-coherence interferometry [30], and coarse spectrum [31]. Most of them provide ~10 nm accuracy in cavity length measurement, which can result in ±5 ℃ error in temperature measurement at around 800 ℃. Additionally, these methods have drawbacks, e.g., limited cavity length range, susceptibility to noise, slow demodulation speed.

To overcome these drawbacks, we proposed and demonstrated a fiber optic temperature sensor based on air-filled FP cavity with variable and controlled pressure [32]. To perform the measurement, the FP reflection spectrum was recorded when the air pressure was varied. Utilizing the fact that the difference of air refractive index and unity is linearly proportional to the pressure and inversely proportional to the absolute temperature, absolute temperature can be extracted from the slope of the spectral shift vs. pressure. The measurement principle dictates that the sensor has zero strain cross-sensitivity over a broad temperature range and the use of air as the sensing material eliminates drifts from material degradation often encountered by solid materials at high temperature. The system demonstrated in our previous work, however, has a few drawbacks that limit its usefulness in practical applications. To achieve a temperature reading, the slope of the spectral shift vs. pressure was obtained using curve fitting by measuring reflection spectrum at multiple air pressure levels over a large pressure range of 1500 psi using a manual air pressure pump. Generating such a high air pressure level is not a trivial task, and often requires expensive and bulky instrumentation, as well as long pumping time.

In this research, we have reported our theoretical and experimental work for further development of the fiber optic temperature sensor toward practical applications. The sensor head is fabricated by splicing a fused-silica capillary tube with a single-mode fiber (SMF) on one end and a side-hole fiber on the other end. The pressure of the cavity is changed by passing air through the holes of the side-hole fiber. Instead of using very high pressure and bulky instruments as in our previous work [32], we used miniaturized instruments with lower pressure (100 psi_g_). Each temperature reading is obtained by recording the spectrum at two pressure levels. This makes the sensor acquisition system compact, portable, fast, and electronically controlled. We developed a model and theoretically analyzed the effect of key system parameters, including the wavelength resolution in the spectral shift and the pressure stability on system performance in terms of temperature measurement resolution and stability. In the experiment, our sensor shows high wavelength resolution (<0.2 pm), linear response, reduced strain cross-sensitivity, and high accuracy with repeatability for a broad operation range (room temperature to over 800 ℃).

## 2. Sensor Fabrication and Theoretical Analysis

### 2.1. Sensor Fabrication

**Figure 1:**
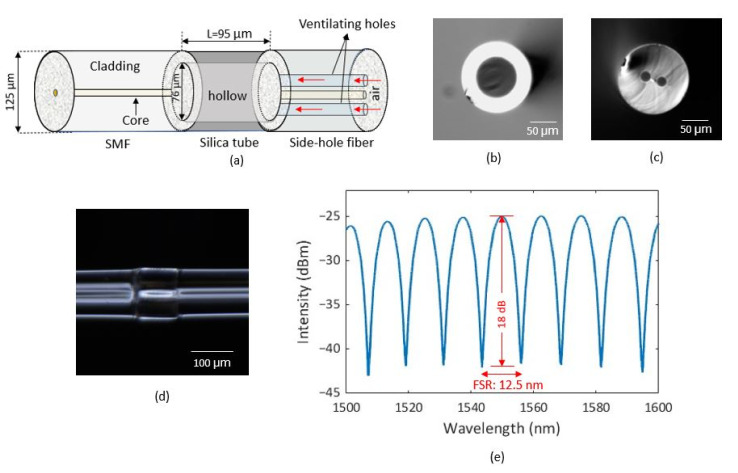
(**a**) Schematic of the sensor; (**b**,**c**) cross-section of silica tube and side-hole fiber, respectively; (**d**) microscopic longitudinal view of the fabricated sensor; (**e**) reflection spectrum of the sensor at ambient temperature and pressure.

Figure 1a shows the schematic of the sensor head, which consists of a single-mode fiber (SMF), a fused-silica capillary tube, and a side-hole fiber of identical diameter (125 µm). The silica tube has a hollow with a diameter of 75 µm inside it. The side hole fiber has two holes with a diameter of ~20 µm along the cladding. Figure 1b,c show a cross-sectional view of the silica tube and side-hole fiber, respectively. Some deformity is seen in the contact edge of cleaver blade. For fabrication, we splice the SMF with silica tube using a fusion splicer using optimized splicing parameters. We cleave the end of the silica tube and splice it again with the side-hole fiber. The length of the silica tube is kept at ~95 µm, and acts as the enclosed FP cavity. Finally, we cleave the side-hole fiber end with an angle of 8° while keeping an appropriate length. The holes in the side-hole fiber are employed as venting holes for pressurizing the FP cavity. The 8° end face of the side-hole fiber effectively eliminates the back reflection from that end face to the SMF. Figure 1d shows the longitudinal view of the fabricated sensor. The reflection spectrum of the sensor at ambient temperature and pressure is shown in Figure 1e. The spectrum shows that the sensor has a good visibility of >18 dB and the full spectral range (FSR) is around 12.5 nm, corresponding to a cavity length of ~96 µm, which is consistent with the silica tube length obtained from the microscope image of the sensor.

### 2.2. Theory and Noise Analysis

The wavelength of a fringe valley of an FPI sensor’s reflection spectrum, λ, is given by:

(1) $$\lambda =\frac{2nl}{m}$$

where *n* is the refractive index of FP cavity material, *l*, is the cavity length, and *m* is the order number of the fringe valley. We used air to fill the FP cavity. Note that l is a function of temperature due to the thermal expansion of tube between the two FP reflectors. It is known that the difference between refractive index of many gases, including the air, and unity (n−1) is, to a good degree of accuracy, proportional to pressure *P*, and inversely proportional to absolute temperature *T* (in the unit of Kelvin), or

(2) $$n-1=\frac{\alpha P}{T}\;$$

where α is a constant characterizing the optical property of the gas and is stable under high temperature. Combining Equations (1) and (2),

(3) $$\lambda =\frac{2l}{m}\left(\frac{\alpha }{T}P+1\right)$$

For temperature measurement, the pressure in the FP cavity (*P*) is varied and the sensor spectrum is measured to determine wavelengths of the fringe valleys as a function of pressure *P*. Specifically, assuming we have obtained the wavelength locations of a fringe valley of a given fringe value order, $\lambda_{1}$ and $\lambda_{2}$, at two different pressure levels of $P_{1}$ and $P_{2}$, respectively, it follows that

(4) $$\lambda_{1}=\frac{2l}{m}\left(\frac{\alpha }{T}P_{1}+1\right)$$

(5) $$\lambda_{2}=\frac{2l}{m}\left(\frac{\alpha }{T}P_{2}+1\right)$$

Solving Equations (4) and (5), we obtain an expression for the temperature independent of the cavity length, *l*, as follows:

(6) $$T=\frac{\alpha \left(P_{2}\lambda_{1}-P_{1}\lambda_{2}\right)}{\lambda_{2}-\lambda_{1}}$$

Note that, in deriving Equation (6), we assumed that the cavity length remains unchanged when the pressure is varied, so the ls (as well as the order number m) in Equations (4) and (5) can be cancelled out. Equation (6) shows that the absolute temperature *T* can be deduced from the two different pressure levels, their corresponding FP interferometric fringe wavelengths, and the parameter α, which is determined by the intrinsic gas property independent of cavity length. Therefore, this method of temperature measurement is not affected by the changes in the cavity length due to thermal expansion or strain. In addition, as gas is generally a stable state at high temperature, α is also stable and the temperature sensor is expected to have little drift.

Equation (6) also indicates that the measurement resolution and accuracy are determined by the resolution and accuracy in determining the fringe valley wavelength and pressure. We theoretically analyzed the effect of the parameters on the system performance. In practice, the system performance would benefit from a large difference between $P_{2}$ and $P_{1}$. Therefore, we can assume $P_{2}\gg P_{1}$, which yields,

(7) $$T\approx \;\frac{\alpha \lambda_{1}P_{2}}{\lambda_{2}-\lambda_{1}}$$

Differentiating (7) with respect to $P_{2}$, we get the resolution (standard deviation) of the temperature measurement, δT, in relation to the resolution of the pressure determination, $\delta P_{2}$,

(8) $$\frac{\delta T}{T}=\frac{\delta P_{2}}{P_{2}}$$

Differentiating (7) with respect to $\lambda_{2}-\lambda_{1}$ and after some simplification, we obtain

(9) $$\frac{\delta T}{T}=\frac{\sqrt{2}\delta \lambda }{\lambda_{2}-\lambda_{1}}$$

where δλ is the resolution of wavelength determination of the fringe valley (which is assumed to be equal for both pressure levels). Equations (8) and (9) show the effect of pressure and wavelength resolutions on temperature measurement resolution, respectively. Since these measurements are independent of each other, we can add both to calculate the resolution of the sensor:

(10) $$\frac{\delta T}{T}=\frac{\delta P_{2}}{P_{2}}+\frac{\sqrt{2}\delta \lambda }{\lambda_{2}-\lambda_{1}}$$

Note that, $\lambda_{2}-\lambda_{1}$, the spectral shift as the pressure changes from $P_{1}$ to $P_{2}$, is also a function of the temperature. Using Equations (1), (4) and (5) and the approximation that n≈1 for air, as well as the assumption that $P_{2}\gg P_{1}$, we obtain $\lambda_{2}-\lambda_{1}\approx \alpha \lambda_{1}P_{2}/T$. Then, we can express Equation (10) as follows:

(11) $$\frac{\delta T}{T}=\frac{\delta P_{2}}{P_{2}}+\left(\frac{\sqrt{2}T}{\alpha P_{2}}\right)\left(\frac{\delta \lambda }{\lambda_{1}}\right)=\frac{\delta P_{2}}{P_{2}}+\left(\frac{\sqrt{2}}{n_{2}-1}\right)\left(\frac{\delta \lambda }{\lambda_{1}}\right)$$

where $n_{2}$ is the air refractive index at pressure $P_{2}$ and temperature T. From Equation (11), it is revealed that the wavelength noise is likely to be the dominant noise contributor to the system noise, because $n_{2}$ is a number close to 1. For example, for dry air at 100 psi and 15 °C, it is estimated that $n_{2}-1\approx 0.002$ at 1550 nm using the value for the air refractive at the standard pressure and the same temperature [33]. The wavelength noise is amplified by 500 times when it is transferred to the measurement noise of temperature. In this work, we used an average wavelength tracking method [34] exploiting multiple valleys in the spectrum to measure λ for varying pressure. This method helps to alleviate the wavelength noise and calculate the sensor resolution more precisely. In addition, the noise contribution from the wavelength noise is proportional to the absolute temperature and inversely proportional to pressure. It means, for a given sensor system with fixed pressure variation, the noise would be dependent on the measurand (temperature) and larger at higher temperature levels.

## 3. Experimental Setup and Sensor Characterization

### 3.1. Experimental Setup

**Figure 2:**
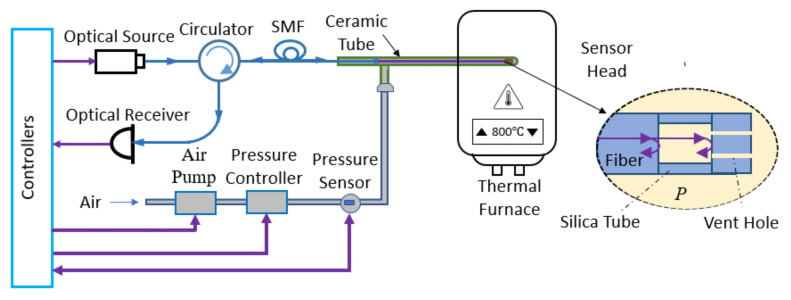
Schematics of the fiber optic temperature sensor system.

**Figure 3:**
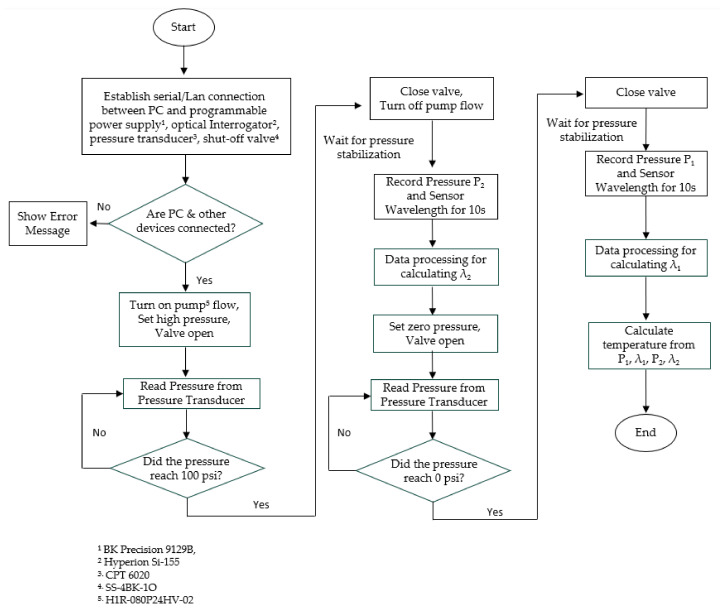
Flowchart of the temperature measurement system algorithm.

We developed a computer-controlled sensor acquisition system with portable and miniaturized instruments. Figure 2 shows the schematic of the system. To measure the sensor’s wavelength spectrum, we used a fiber optic sensor interrogator (Model: Hyperion si-155, manufacturer: Luna Innovations, VA, USA) with 1 pm accuracy. We used a thermal furnace to increase the temperature for sensor characterization. The pressure in the FP cavity is changed by a miniaturized electronic pressure pump (model: H1R-080P24HV-02, Manufacturer: Parker Hannifin, NH, USA). For our experiment, we chose two different pressure levels of 0 psi_g_ and 100 psi_g_ (or 690 kPa) as $P_{1}$ and $P_{2}$, respectively. We used an electrical shut-off valve to stabilize the pressure fluctuations while taking the readings. To read the pressure, we used a precision pressure transducer with 0.020% full-scale accuracy. The pump, valve, and pressure transducer are miniaturized in size (longest dimension is <4.3 inch) and electronically controlled. We used a programmable power supply to run these instruments. The whole setup is programmed in MATLAB code and the wavelength and pressure data are stored simultaneously in the control unit (computer). It takes less than one minute to pressurize the chamber from 0 psi_g_ to 100 psi_g_ and the pressure becomes stable very quickly after shutting the valve off. We used an alumina ceramic tube to house the sensor in a pneumatic setup to prevent any air leak. Additionally, ceramic tubes have high melting temperature (over 2500 ℃) and flexural strength (~54,000 psi), which makes it suitable for high-temperature applications. As described in the theory section, temperature can be measured by taking the data of two pressure levels $P_{1}$ and $P_{2}$, their corresponding wavelengths $\lambda_{1}$ and $\lambda_{2}$. Figure 3 shows a flowchart of the integrated computer-controlled temperature measurement algorithm.

### 3.2. Spectral Shift with Pressure

**Figure 4:**
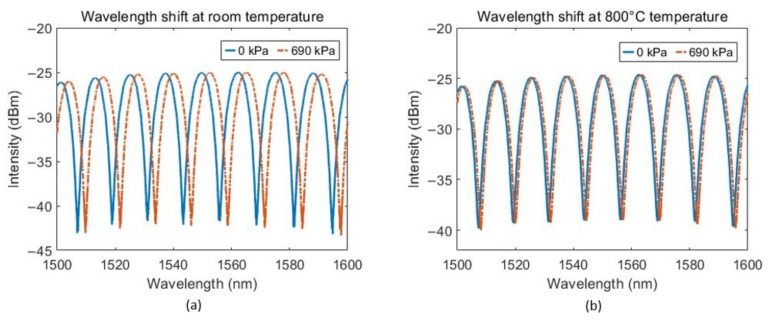
Reflection spectra for two different pressure levels at (**a**) room temperature and (**b**) 800 ℃ temperature.

**Figure 5:**
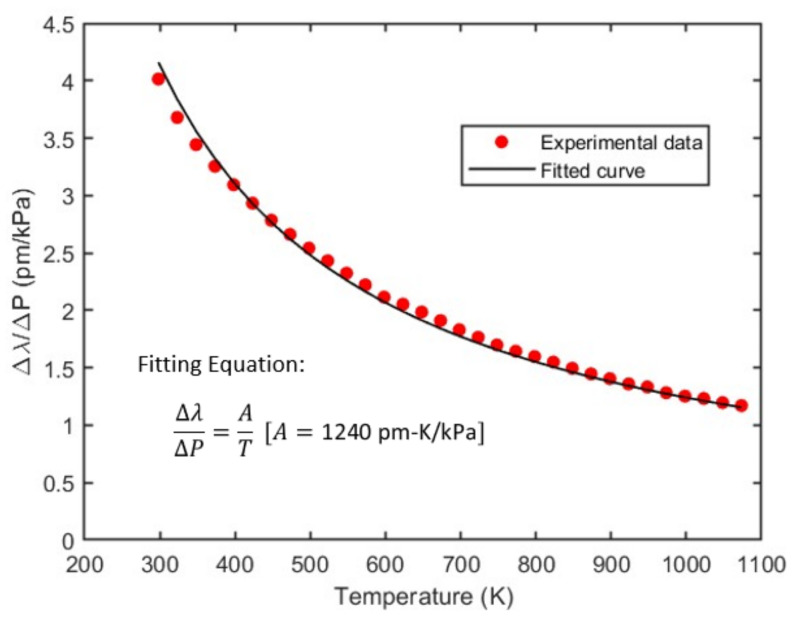
Spectral shift with pressure at different temperatures.

Using the above setup, we increased the pressure from 0 to 100 psi or 690 kPa (pressure is relative to gauge, not absolute pressure) to see the spectral shift of the sensor. It was seen that the interferometric fringes shift toward the higher wavelength with increase in pressure. This shift was reduced at high temperature. For example, the spectral shift with pressure decreases from 4.01 pm/kPa to 1.17 pm/kPa when the temperature is increased from room temperature (25 ℃) to 800 ℃ (see Figure 4). To see how the spectral shift is affected at different temperatures, we increased temperature from 25 ℃ to 800 ℃ with step size of 25 ℃ and recorded the wavelength shift for 690 kPa pressure change. Figure 5 shows the graph of spectral shift per unit pressure change (Δλ/ΔP) vs. absolute temperature (T), where Δλ is the spectral shift for a change in pressure ΔP. This curve is fitted with the theoretical curve. Subtracting Equation (4) from Equation (5) yields

(12) $$\frac{\lambda_{2}-\lambda_{1}}{P_{2}-P_{1}}=\frac{\Delta \lambda }{\Delta P}=\frac{2l}{m}\alpha \approx \frac{\lambda \alpha }{T}=\frac{A}{T}$$

Here, A=λα and λ is the central wavelength, which is used for cavity length calculation. Fitting the experimental results with Equation (12) leads to the fitting parameter, A= 1240 pm-K/kPa and shows a good agreement between the fitting curve and the experimental results, as shown in Figure 5, indicative of the validness of the theoretical model. This also experimentally validated that the gas material properties α have negligible effects over this broad temperature range, and can be considered as constants in our proposed model.

### 3.3. Wavelength Resolution

**Figure 6:**
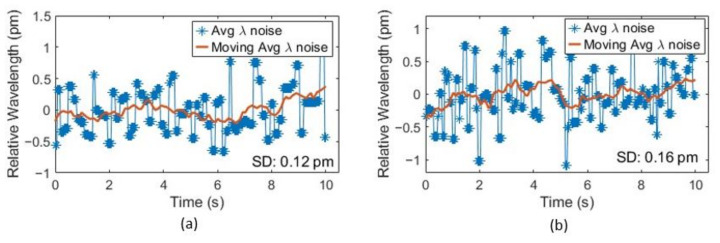
Sensor wavelength resolution at room temperature for pressure of (**a**) 0 psi_g_ and (**b**) 100 psi_g_.

**Figure 7:**
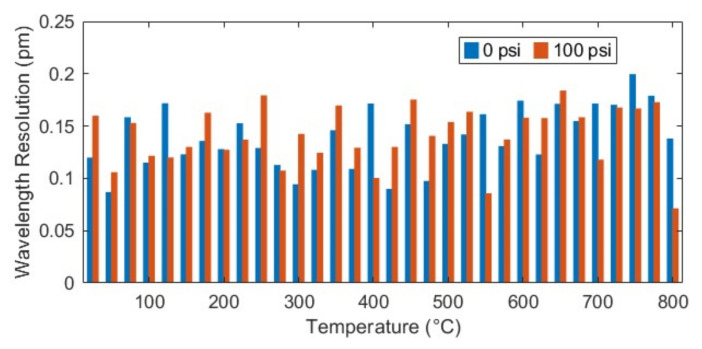
Sensor wavelength resolution for two different pressure levels at each measurement.

Wavelength resolution is defined as the variation in the fringe wavelength over a small period. It is calculated by taking the standard deviation of the moving average of multiple valley wavelengths. For this, we recorded the reflection spectrum from the fiber optic sensor interrogator for 10 s of duration at 1 kHz scanning rate. Instead of using only one fringe valley as in our previous work [32], we took the arithmetic average of the first seven valleys in the range of 1500–1600 nm to find the parameter $\bar{\lambda }$ for a specific temperature and pressure. This method eliminates the wavelength noise caused by spurious jumps in any specific valley by a factor of $\sqrt{M}$, where *M* is the number of valleys (or peaks) used to calculate average wavelength [34]. Since we recorded data for a duration of 10 s for each measurement, the moving average of $\bar{\lambda }$ is taken to calculate the wavelength λ to be used in Equation (6). Figure 6 shows that the sensor resolution at ambient room temperature (25 ℃) is 0.12 pm and 0.16 pm for two pressure levels, 0 and 100 psi, respectively. To measure the sensor resolution at high temperature, we continued the same test from 25 ℃ to 800 ℃. Figure 7 shows the wavelength resolution of the sensor at different temperatures. It can be seen that the wavelength resolution was found to be in the range of 0.07–0.2 pm even with this broad operation range. According to Equation (10), the accuracy for temperature measurement with this resolution is theoretically within ±0.32 ℃.

### 3.4. Pressure Fluctuation

**Figure 8:**
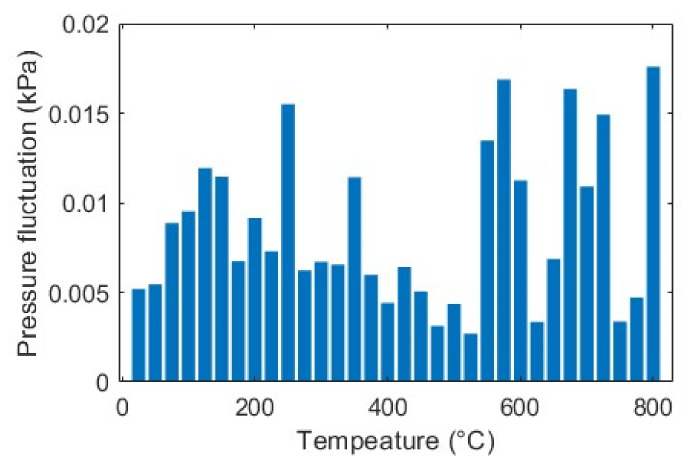
Pressure fluctuation $\delta P_{2}$ at each measurement.

As described earlier, pressure stability is another prerequisite for our sensor’s accurate temperature measurement. Theoretically, the fluctuation in high pressure $P_{2}$, (for our case, 690 kPa) can cause the temperature measurement error by $\delta P_{2}/P_{2}$ (Equation (9)). We measured the pressure of the system using a precision pressure transducer (CPT6020) while performing the wavelength resolution test. Figure 8 shows the pressure fluctuations $\delta P_{2}$ for different measurements. It can be seen that the system is quite stable, limiting the pressure fluctuations in the range of $\left(0.0027\sim 0.0176\right)$ kPa. With this setup, the temperature measurement error due to this pressure fluctuation is negligible (<0.027 ℃).

### 3.5. Linearity Test

**Figure 9:**
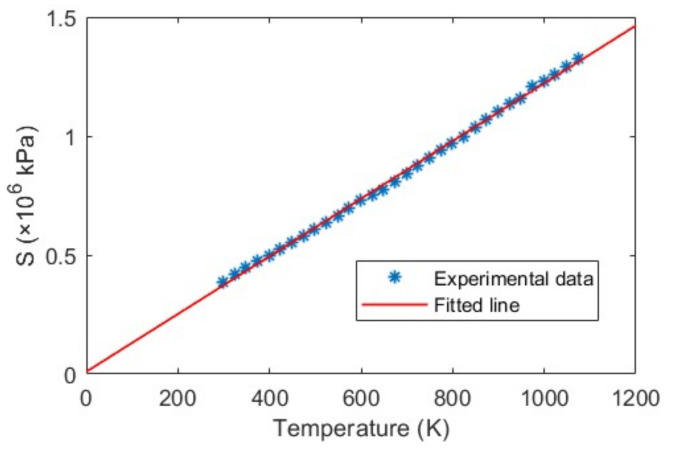
Linear relationship between temperature *T* and *S*.

To validate our proposed temperature measurement method according to Equation (6), we calculated $S=\left(P_{2}\lambda_{1}-P_{1}\lambda_{2}\right)/\left(\lambda_{2}-\lambda_{1}\right)$ for different temperatures ranging from 25 ℃ to 800 ℃. Figure 9 shows the linear relationship between the absolute temperature *T* (in K unit) and *S* (in kPa). The R-square of the linear fitting coefficient is found 0.9986. This shows that the sensor is linear up to a high temperature over at least 800 ℃. The good agreement is also an indication of the validity of the theoretical model. From Equation (6), the value of the gas (air) constant, α is found $8.2515\times 10^{-4}$ K/kPa (or 0.00568 K/psi), which matches our previous work [32]. Therefore, this temperature measurement method requires no calibration even if the sensor’s cavity length is different.

### 3.6. Repeatability and High-Temperature Test

To investigate the repeatability of our fabricated sensor, we performed a thermal-cycle test in which we repeatedly heated the sensor from room temperature to 800 ℃ and then cooled the sensor again to room temperature. In each cycle, we measured the room temperature and high temperature according to Equation (6) by using the empirical value of gas constant $\alpha =8.2515\times 10^{-4}\;K/\mathrm{kPa}$. We also measured the temperature each time with a reference thermometer (Pt100) with a high accuracy of ±0.1 ℃ for comparison. Table 1 shows that temperature measured by our sensor matches well with the reference thermometer to a high degree of accuracy. At room temperature, the difference is ~0.5 ℃, whereas at a high temperature of 800 ℃, it increased to ~3 ℃. This can be explained by the fact that at this high temperature, the furnace has limitations with respect to accuracy (±3 °C) and temperature uniformity (±4.8 °C), and the accuracy of the reference thermocouple can also deteriorate. The thermal cycle test proves that our fabricated sensor’s accuracy is repeatable even after heating it to a very high temperature several times. The excellent accuracy from the thermal cycle test is also proof of the validity of the theoretical model of the sensor operation.

## 4. Discussion

Fiber optic FP temperature sensors have become a widely explored research topic during the last few decades. Most of the sensors use solid material (e.g., glasses, polymers, silicon, etc.) as the sensing element, and have inherent limitations with respect to high-temperature measurement. Many solid materials have a low temperature capability. Their optical properties and sensor structures may also undergo permanent changes at high temperature that lead to sensor signal drift. The uniqueness of the fiber optic temperature sensor studied here is the usage of air as the sensing material and a sensing mechanism that leads to high stability at high temperature. The sensor consists of an FP cavity filled with air whose pressure can be controlled. It utilizes the fact that the difference in air refractive index and unity is proportional to the air pressure and inversely proportional to the air absolute temperature. The optical properties of air are stable at high temperature. The absolute temperature is obtained by measuring the spectra of the FP reflection fringes at two different pressure levels, which is independent of the cavity length. Therefore, even if the enclosure (a glass tube in this case) of the air in the FP cavity were to change its length or undergo permanent deformations at high temperature, it would not affect the accuracy of the sensor.

An assumption we need to make for the sensor to work is that the temperature and the cavity length remain unchanged when the pressure is changed. Therefore, in practical applications, it is critical to ensure that the sensor spectra are measured at two pressure levels within a short time over which the ambient temperature and the cavity length exert negligible influence. In practical applications, the pressure can reach equilibrium within a few seconds and high-speed spectrometers can be used for spectrum measurement, which means that the temperature and the FP cavity need to be stable over a period of a few seconds.

The use of the gas pressure system increases the size of the sensor system. It also constrains the distance between the sensor head and the pressure devices. In this work, we developed an electronically controlled system with miniaturized and precision devices, making the measurement more practical and ensuring good accuracy and stability. This method can be used in many applications that require a stable and accurate temperature sensor, for example, temperature measurement in coal-gasified power plants.

We also analyzed the contribution of these parameters in temperature measurement deviation. Specifically, we found that the relative wavelength resolution in determining the wavelength shift is the main noise source of the system and its noise contribution is also a function of the temperature itself. This analysis is important for the system design to achieve optimized system performance.

## 5. Conclusions

In this work, we demonstrated an FP interferometer temperature sensor system based on the spectral shift of fringe wavelength due to the pressure change in FP cavity. The sensor fabrication process is very straightforward, and involves splicing a fused-silica tube with a regular SMF and a side-hole fiber. Air is passed through the holes of the side-hole fiber to change the cavity pressure up to 100 psi by using a miniaturized pump. The whole pressure calibration and sensor acquisition system is electronically controlled, miniaturized and fast. In addition, we developed a model to analyze the impact of the system key parameters, such as sensor wavelength resolution and pressure fluctuation, on temperature measurement. In the experiment, the sensor was characterized from room temperature to 800 ℃ using our setup. The sensor’s wavelength resolution was found to be less than 0.2 pm, with a high pressure stability (~0.015 kPa). According to our model, this resolution and pressure fluctuation contribute to a temperature measurement error of only 0.32 ℃, which is excellent compared to other fiber optic sensors, especially for this broad range of temperatures. The repeatability of the sensor was tested by subjecting it to several thermal cycles from room temperature to 800 ℃ and comparing the measurements to a high-accuracy reference thermometer. The comparison demonstrates the high accuracy of the sensor even after multiple cycles.

## Figures and Tables

**Table 1:** Comparison of temperature measurement by our sensor and a reference thermocouple.

Room Temperature Comparison			High Temperature Comparison		
Reference Sensor ^1^ Temperature (°C)	Fabricated Sensor Temperature (°C)	Difference (°C)	Reference Sensor ^1^ Temperature (°C)	Fabricated Sensor Temperature (°C)	Difference (°C)
21.90	22.42	0.52	799.50	796.97	2.53
22.90	23.15	0.25	799.80	800.25	−0.45
21.70	22.11	0.41	799.20	800.64	−1.44
21.60	22.11	0.51	798.60	795.85	2.75
26.20	26.32	0.12	798.90	796.71	2.19
26.00	26.20	0.20	799.00	802.01	−3.01
23.20	23.22	0.02	800.60	797.77	2.83

^1^ Pt100 is used as reference sensor.

## Data Availability

The data presented in this study are available on request from the corresponding author.

## Disclaimer

This report was prepared as an account of work sponsored by an agency of the United States Government. Neither the United States Government nor any agency thereof, nor any of their employees, makes any warranty, express or implied, or assumes any legal liability or responsibility for the accuracy, completeness, or usefulness of any information, apparatus, product, or process disclosed, or represents that its use would not infringe privately owned rights. Reference herein to any specific commercial product, process, or service by trade name, trademark, manufacturer, or otherwise does not necessarily constitute or imply its endorsement, recommendation, or favoring by the United States Government or any agency thereof. The views and opinions of authors expressed herein do not necessarily state or reflect those of the United States Government or any agency thereof.
